# EphB2 knockdown decreases the formation of astroglial‐fibrotic scars to promote nerve regeneration after spinal cord injury in rats

**DOI:** 10.1111/cns.13641

**Published:** 2021-04-01

**Authors:** Jian Wu, Bing Lu, Riyun Yang, Ying Chen, Xue Chen, Yi Li

**Affiliations:** ^1^ Department of Histology and Embryology Medical School Nantong University Nantong China; ^2^ Wuxi Medical School Jiangnan University Wuxi China

**Keywords:** astroglial‐fibrotic scar, EphB2, RNAi, spinal cord injury

## Abstract

**Aims:**

At the beginning of spinal cord injury (SCI), the expression of EphB2 on fibroblasts and ephrin‐B2 on astrocytes increased simultaneously and their binding triggers the formation of astroglial‐fibrotic scars, which represent a barrier to axonal regeneration. In the present study, we sought to suppress scar formation and to promote recovery from SCI by targeting EphB2 in vivo.

**Methods:**

The female rats SCI models were used in vivo experiments by subsequently injecting with EphB2 shRNA lentiviruses. The effect on EphB2 knockdown was evaluated at 14 days after injury. The repair outcomes were evaluated at 3 months by electrophysiological and morphological assessments to regenerated nerve tissue. The EphB2 expression and TGF‐β1 secretion were detected in vitro using a lipopolysaccharides (LPS)‐induced astrocyte injury model.

**Results:**

RNAi decreased the expression of EphB2 after SCI, which effectively inhibited fibroblasts and astrocytes from aggregating at 14 days. The expression of EphB2 in activated astrocytes, in addition to fibroblasts, was significantly increased after SCI in vivo, in line with upregulated expression of EphB2 and increased secretion of TGF‐β1 in astrocyte culture treated with LPS. Compared to the scramble control, RNAi targeting with EphB2 could promote more nerve regeneration and better myelination.

**Conclusions:**

EphB2 knockdown may effectively inhibit the formation of astroglial‐fibrotic scars at the beginning of SCI. It is beneficial to eliminate the barrier of nerve regeneration.

## Graphical abstract

Pattern of astroglial‐fibrotic scars formation: The activated astrocytes express ephrin‐B2 also highly express EphB2, which maybe participate in the formation of glial scars. Moreover, the activated astrocytes expressing ephrin‐B2 and the fibroblasts expressing EphB2 together form the astroglial‐fibrotic scars. Therefore, EphB2 knockdown could inhibit the formation of glial scars and astroglial‐fibrotic scars.

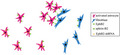

## 1 INTRODUCTION

Spinal cord injury (SCI) is generally considered to be an irreversible process.^1^ One major factor is the rapid appearance of the astroglial‐fibrotic scar, which is composed of fibronectin (FN)‐positive lesion core surrounded by a glial fibrillary acidic protein (GFAP)‐positive lesion border.^2^, ^3^ The astroglial‐fibrotic scar is a mechanical barrier to axonal regeneration, and it secretes chondroitin sulfate proteoglycans (CSPGs), which is known to negatively affect neurite growth.^4^ Therefore, various methods for removing astroglial‐fibrotic scars have been reported, such as degradation of CSPGs by chondroitinase ABC,^5^ decomposition of keratan sulfate by keratanase,^6^ inhibition of the accumulation of CSPGs using high‐molecular‐weight hyaluronic acid,^7^ knocking out of GFAP and vimentin genes,^8^ and photochemical removal of astroglial‐fibrotic scars.^9^ However, these measures only degrade some of the components of astroglial‐fibrotic scars that have already formed, and thus, the effect is limited. Therefore, it would be more effective to reduce the formation of astroglial‐fibrotic scars from the beginning.

Many studies have shown that the formation of astroglial‐fibrotic scars is the result of the interaction of multiple cells,^10^ including astrocytes and fibroblasts.^11^, ^12^ Bundesen and colleagues have confirmed that at the beginning of SCI, the expression of the receptor EphB2 on fibroblasts is significantly upregulated, triggering the formation of astroglial‐fibrotic scars by binding to the corresponding ligand ephrin‐B2, which is upregulated in astrocytes simultaneously.^13^ When fibroblasts and astrocytes aggregate, a compact cell boundary near the injury border is formed and the gaps between the cells get filled by extracellular matrix, such as CSPGs.^14^, ^15^ Two weeks after injury, the astroglial‐fibrotic scar is formed at the injury border. Although other types of cells are involved in the formation of astroglial‐fibrotic scars,^16^, ^17^ the accumulation of fibroblasts and astrocytes at the injury border mediated by upregulation of EphB2 and ephrin‐B2 is the basis for the scar formation.^18^, ^19^ Therefore, specific inhibition of this upregulation may inhibit the effective formation of astroglial‐fibrotic scars and may be another way for promoting SCI repair.

The expression of EphB2 in the human pancreatic cancer cell line, CFPAC‐1, was successfully suppressed by RNAi to elucidate the relationship between EphB2 and tumor formation.^20^ Ephrin‐B2 expression was successfully reduced by injecting specific shRNA targeted *ephrin*‐*B2* to relieve neuropathic pain.^21^ Therefore, we used RNAi to specifically inhibit the increased expression of EphB2 after SCI. Its binding to the corresponding ephrin‐B2 decreased, thus effectively preventing aggregation of fibroblasts and astrocytes. This may decrease the formation of astroglial‐fibrotic scars, facilitate the regeneration of axons across the damaged zone, and promote the recovery of the morphology and electrical conduction of the injured spinal cord.

## 2 MATERIALS AND METHODS

### 2.1 RNAi knockdown

A lentiviral vector (pLV‐shRNA‐zsGreen1, see Figure S1) containing shRNA (Biomics Biotechnologies, Nantong, China) that targets the *EphB2* and the *ZsGreen1* reporter gene was injected into rats for the in vivo experiment. The positive‐sense strand sequence of rat‐Ephb2‐sh‐2 was GATCCAGAAGGAGCUCAGUGAGUAdTdTTTTTTT, and the antisense was GUACUCACUGAGCUCCUUCUdTdTAAAAAA. The scrambled shRNA non‐silencing control sequence was GAGTAAGAACGTAGCGAGT. The virus titer was 1.5 × 10^9^ TU/ml.

### 2.2 Animals and surgery

Adult female specific pathogen‐free Sprague–Dawley (SD) rats (11–12 weeks old, 200–220 g) were purchased from the Experimental Animal Center of Nantong University (Nantong, China). All surgical interventions and postoperative animal care were performed in accordance with the Institutional Animal Care guidelines and received ethical approval from the Administration Committee of Jiangsu Province of China.

SCI animal model preparation: To examine the changes in mRNA expression of *EphB2* and *ephrin*‐*B2* after SCI, the rats were divided into a 0 day group and an injury group observed at 2, 4, 7, 14 days and 3 m. In the RNAi experiment, animals were divided into three groups: Sham group, specific RNAi group (SCI+EphB2 shRNA group), and non‐specific RNAi group (SCI+cont shRNA group). Establishment of the SCI animal model and construction of the lentiviral vector containing shRNA injection were based on a method published by Donnelly et al.^22^ Details are provided in Supplementary Materials and Methods.

### 2.3 In vitro lipopolysaccharide (LPS)‐induced astrocyte injury model

Primary astrocyte cultures were prepared from spinal cords of SD rats at postnatal day 1 as previously described.^23^ After 48 h of LPS treatment (10 µg/ml in saline; Sigma), the culture medium was collected to examine the TGF‐β1 levels using an ELISA kit (Abcam) according to the manufacturer's instructions. Saline treatment was used as the control group. Furthermore, the cells were collected to determine the EphB2 and ephrin‐B2 protein levels using Western blot analysis.

### 2.4 Quantitative PCR and Western blot analysis

Fresh spinal cords from the different groups were acquired from the rats under anesthesia at 2 mm of the cranial and caudal levels from the center of injury at the indicated time points. RNA and protein were extracted. For subsequent procedures, see . PCR primers (Table S1) were synthesized by Invitrogen. Antibodies used for Western blot were seen in Table 1.

**TABLE 1:** Primary and secondary antibodies used in this study

	Antibody	Manufacturer	Product code	Dilution
Western blot	Rabbit anti‐EphB2 IgG	Cell Signal	83029	1:1000
	Rabbit anti‐ephrin‐B2 IgG	Abcam	ab150411	1:1000
	Mouse anti‐β‐actin IgG	Sigma	A5441	1:5000
	HRP conjugated goat anti‐rabbit IgG	Sigma	A0545	1:5000
	HRP conjugated goat anti‐mouse IgG	Sigma	A3682	1:5000
Immunohistochemistry	Rabbit anti‐EphB2 IgG	Cell Signal	83029	1:200
	Mouse anti‐GFAP IgG	Cell Signal	3670	1:300
	Rabbit anti‐GFAP IgG	Cell Signal	12389	1:200
	Goat anti‐GFAP IgG	Abcam	Ab53554	1:500
	Rabbit anti‐ephrin‐B2 IgG	Abcam	ab150411	1:200
	Rabbit anti‐FN IgG	Abcam	ab45688	1:200
	Mouse anti‐FN IgG	Abcam	Ab6328	1:200
	Rabbit anti‐neurofilament‐H	Abcam	Ab207176	1:500
	Mouse anti‐neurofilament‐H	Cell Signal	2836	1:400
	Alexa Fluor® 568‐conjugated donkey anti‐mouse IgG	Thermo Scientific	A10037	1:600
	Alexa Fluor® 568‐conjugated donkey anti‐rabbit IgG	Thermo Scientific	A10042	1:600
	Alexa Fluor® 568‐conjugated donkey anti‐goat IgG	Thermo Scientific	A11057	1:600
	Alexa Fluor® 647‐conjugated donkey anti‐mouse IgG	Thermo Scientific	A31571	1:600
	Alexa Fluor® 647‐conjugated donkey anti‐rabbit IgG	Thermo Scientific	A31573	1:600
	Alexa Fluor® 647‐conjugated donkey anti‐goat IgG	Thermo Scientific	A21447	1:600

### 2.5 Morphological assessment

The rats were perfused with 4% paraformaldehyde solution, and the spinal cords were severed 5 mm from each end of the injection point outwards. Samples were fixed, dehydrated by gradient sucrose, and transected serially using a freezing microtome (CM1900 Leica) at a thickness of 16 µm.

Conventional immunofluorescence: The samples were blocked and permeabilized and then incubated with primary antibodies (Table 1) overnight at 4°C. After three washes with PBS, the sections were incubated with secondary antibodies (Table 1) at 37°C for 2 h. Hoechst labeling was used to visualize the nuclei. Samples were observed and recorded under the corresponding excitation wavelengths using fluorescence microscopy (DM4000B, Leica) or laser confocal microscopy (SP7, Leica). A slice was collected every 10 sections; the slices containing the injury area (about 30–35 slices per rat spinal cord) were stained for immunofluorescence histochemistry; and the positive staining area and the fluorescence intensity were analyzed by an image analysis system (Leica QWin V3). Quantitative analysis was carried out in a double‐blinded manner.

Transmission electron microscopy: Three months after SCI, ultrathin sections were cut at injury site (IS) and caudal spinal cord (CS) (shown in Figure 6 upper right inset) and stained with lead citrate and uranyl acetate. Samples were observed under a transmission electron microscope (JEOL).

### 2.6 Electrophysiological assessment

Three months after SCI, the motor evoked potentials (MEPs) were recorded. Briefly, after the rats were anesthetized, the stimulating electrode (60 mA in strength) was placed under the skin of the rat's head, and the recording electrode was placed on the calf muscle. The amplitude and incubation period of MEP were obtained.

### 2.7 Statistical analysis

Data analysis and statistical analysis were performed by SPSS 19.0. Levene's test was used to assess data normality. All data were analyzed by one‐way ANOVA plus Dunnett's post hoc test for multi‐group comparisons or with Student's *t* test for two‐group comparisons. Data are expressed as mean ± standard deviation (SD). *p* < 0.05 was considered statistically significant.

## 3 RESULTS

### 3.1 Effect of EphB2 and ephrin‐B2 expression by RNAi

**FIGURE 1:**
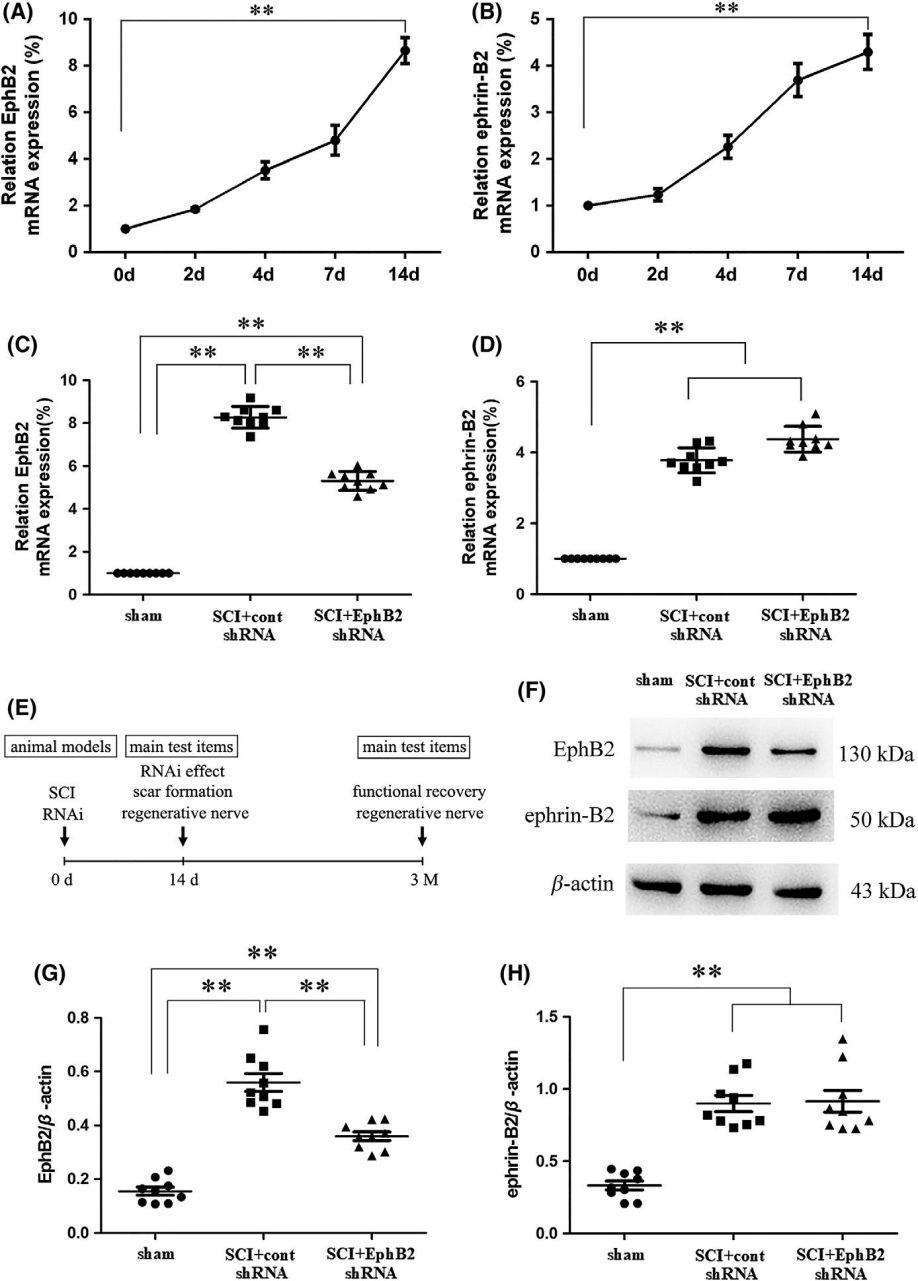
EphB2 and ephrin‐B2 expression after SCI at day 14. The mRNA expression of EphB2 (A) and ephrin‐B2 (B) increased continuously with time after SCI; it increased by more than 8‐fold and 4‐fold compared with 0 day group on day 14, respectively (**p < 0.01, one‐way ANOVA, *n* = 8 or 9 at each time point). After RNAi, the high expression of EphB2 mRNA was inhibited at 14 days after SCI+RNAi (C, *n* = 9). The high expression of ephrin‐B2 mRNA after RNAi was not affected (D, *n* = 9). The experimental timeline shows the main items tested at 14 days and 3 months after SCI (E). Fourteen days after SCI, injured spinal cords were collected for Western blot assay to determine the expression of EphB2 and ephrin‐B2 (F). Histogram showed the expression of EphB2 (G) and ephrin‐B2 (H) proteins (*n* = 9, respectively). **p < 0.01, one‐way ANOVA

Bundesen et al^13^ have reported the high expression of EphB2 and ephrin‐B2 protein after SCI. We used qPCR to examine the change in mRNA expression of *EphB2* and *ephrin*‐*B2* after SCI. Consistent with the abovementioned study, with time there was a progressive expression increase until 14 days after SCI (Figure 1A,B).

As the astroglial‐fibrotic scar is formed maturely at 14 days after SCI, we selected 14 days as the observation point for the formation of astroglial‐fibrotic scar by RNAi, and functional recovery was detected at 3 months (Figure 1E). The EphB2 mRNA and protein expression levels at the injury sites and the surrounding tissues in the cont shRNA group were significantly higher than those in the Sham group (*p* < 0.01; Figure 1C,F,G) 14 days after injury. EphB2 RNAi (SCI+EphB2 shRNA group) significantly reduced the high expression of EphB2 mRNA and protein after injury (*p* < 0.01) compared with the cont shRNA group (Figure 1C,F,G). In addition, injection of EphB2 shRNA after SCI had no effect on the high expression of ephrin‐B2 at the mRNA and protein levels (Figure 1D,F,H). These data indicated that the RNAi injection technique effectively reduced the high expression of EphB2 at the site of injury after SCI.

### 3.2 Histological evaluation of astroglial‐fibrotic scar formation and NF expression by RNAi 14 days after SCI

**FIGURE 2:**
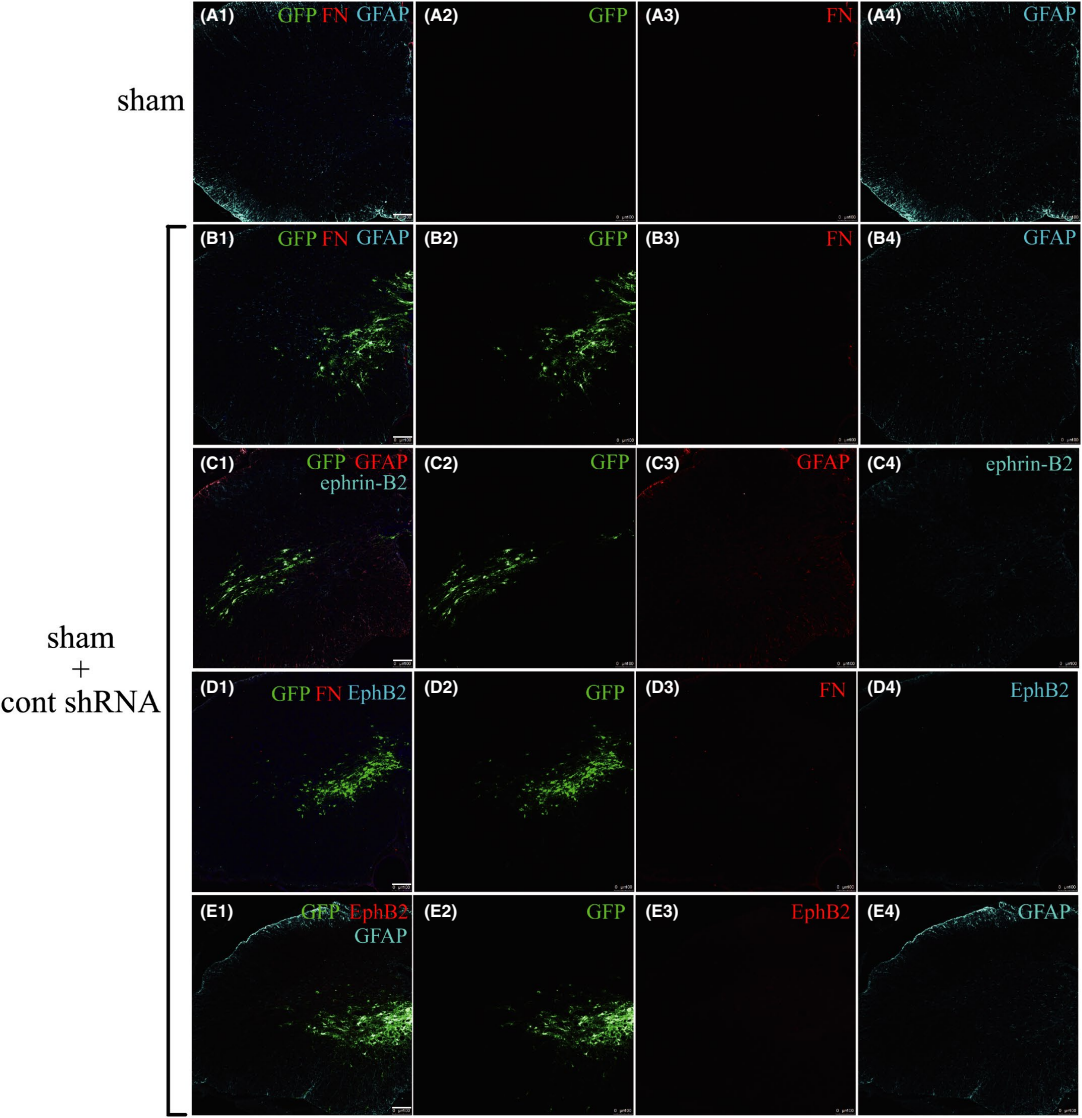
The immunofluorescent technique showed no fibroblasts that express FN and normal morphological astrocytes that express GFAP in sham group (A1–A4) and in SCI+cont shRNA group (B1–B4). The immunofluorescent technique showed normal GFAP and normal ephrin‐B2 in sham + cont shRNA group (C1–C4). The immunofluorescent technique showed no FN and no EphB2 in sham + cont shRNA group (D1–D4). The immunofluorescent technique showed no EphB2 and normal GFAP in sham + cont shRNA group (E1–E4). Scale bar = 100 μm

Lentiviral vectors containing the cont shRNA were injected into the spinal cord in the Sham+cont shRNA group. The fluorescence of cells expressing GFP diffused from the injection point at 14 days. As with the Sham group (Figure 2A1–4), no activated astrocytes were found in the spinal cord, except for the injection point, and there was no astrocytes and fibroblasts aggregation in the Sham+cont shRNA group (Figure 2B1–4). These results indicated that the lentiviral vector caused no damage to the spinal cord cells and that the shRNA successfully entered the cells and was expressed.

**FIGURE 3:**
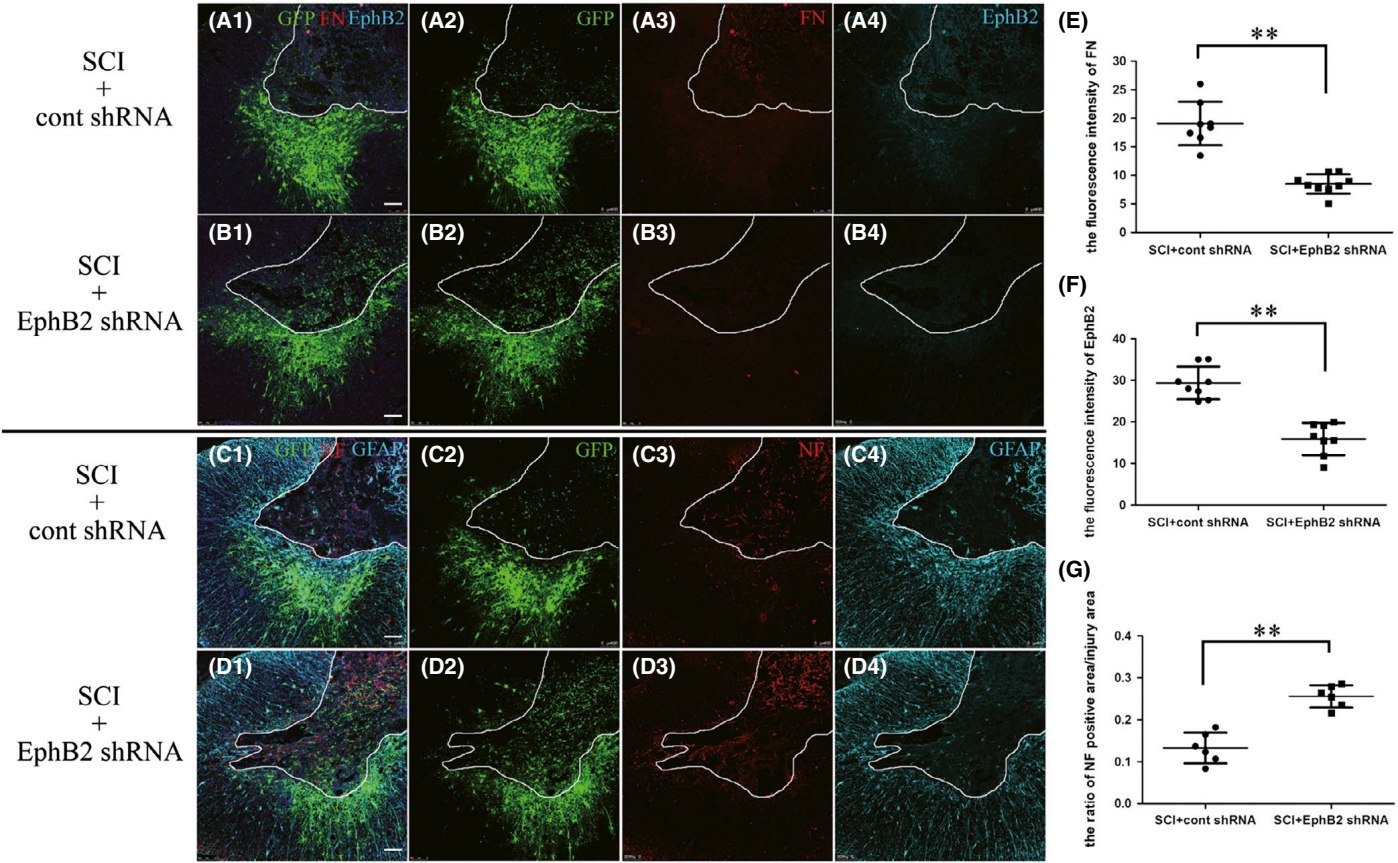
The expression of FN, EphB2, GFAP, and NF after SCI at day 14. The immunofluorescence technique showed the expression of FN and EphB2 in the SCI+cont shRNA group (A1–A4) and the SCI+EphB2 shRNA group (B1–B4). The fluorescence intensity of FN and EphB2 at the injury site showed a significant difference between the two groups (E and F, respectively; *n* = 8 or 9). The immunofluorescence technique showed the expression of NF and GFAP in the SCI+cont shRNA group (C1–C4,) and the SCI+EphB2 shRNA group (D1‐D4). The NF‐positive area/injury area ratio showed a significant difference at the injury between the two groups (G, *n* = 6). The injuries area is enclosed by solid lines. Scale bar = 100 µm, **p < 0.01, *t*‐test

EphB2 is mainly expressed in fibroblasts; however, as there were very few fibroblasts in the normal spinal cord (Figure 2A3,B3,D3), EphB2 immunofluorescence was not detected in the Sham+cont shRNA group (Figure 2D1–4). In contrast, fibroblasts clustered at the injury site 14 days after SCI (Figure 3A1–4); thus, the expression of EphB2 significantly increased at the same region (Figure 3A1–4). The fluorescence intensity of FN and EphB2 (Figure 3B1–4) was significantly decreased at the injury site after RNAi (*p* < 0.05, Figure 3E,F, respectively). Fourteen days after SCI, a large number of activated astrocytes that expressed GFAP showed significant hyperplasia, hypertrophy, and dense aggregation in the region surrounding the injury in the SCI+cont shRNA group (Figure 3C1–4). However, the astrocytes were still activated but did not aggregate at the edge of the lesion after RNAi (Figure 3D1–4). The neurofilament (NF) immunofluorescence showed the regenerative axons in the injury area (Figure 3C3,D3). The ratio of NF area to the injury area in the SCI+EphB2 shRNA group was significantly higher compared with that in the SCI+cont shRNA group (*p* < 0.01; Figure 3G). These data suggested that there were more regenerative axons growing into the injury area after RNAi.

### 3.3 ephrin‐B2 and EphB2 expression in astrocytes

**FIGURE 4:**
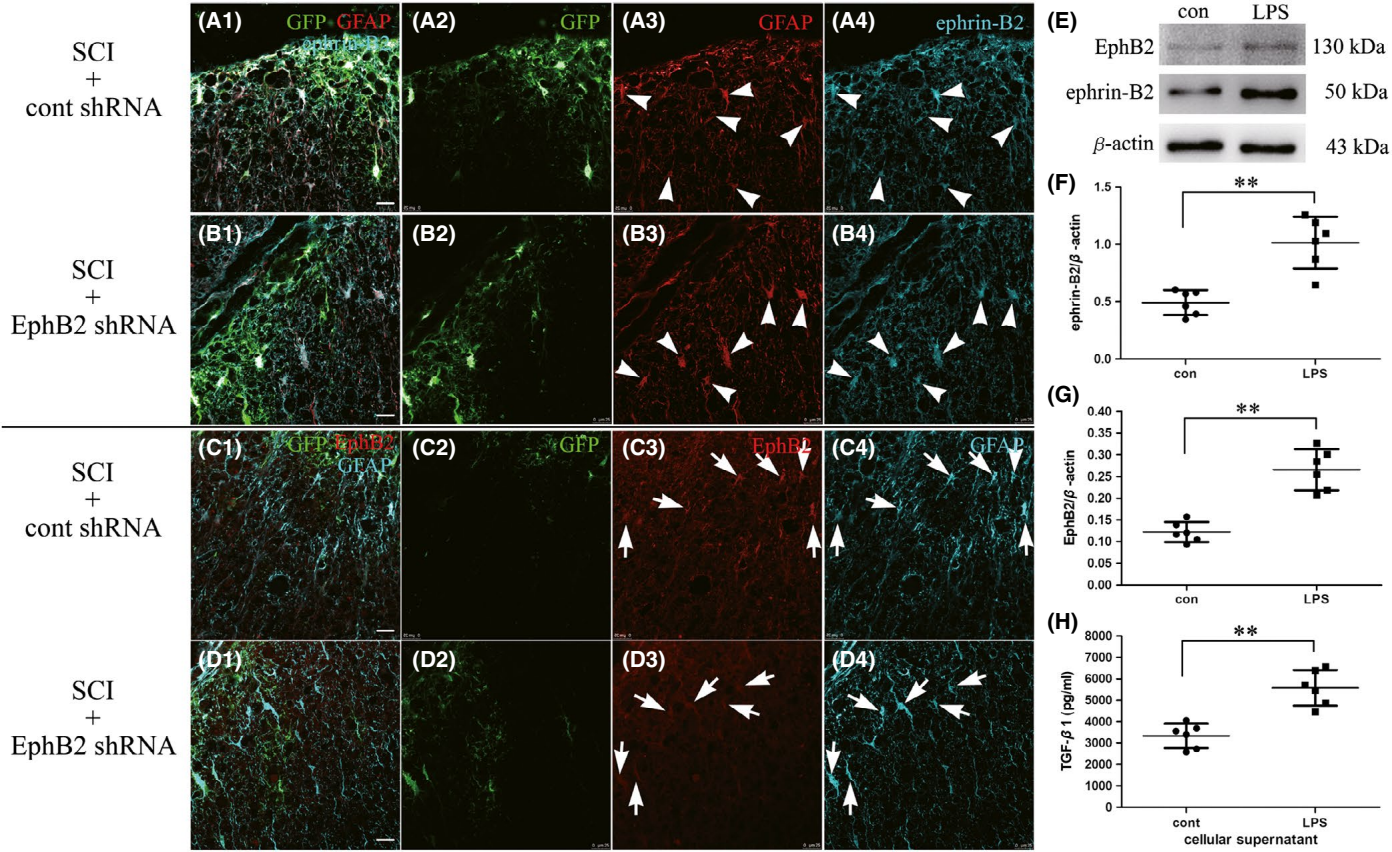
The expression of ephrin‐B2 and EphB2 on the activated astrocytes. The immunofluorescent technique showed higher expression of ephrin‐B2 on the activated astrocytes both in SCI+cont shRNA group (A1–A4) and SCI+EphB2 shRNA group (B1–B4) after SCI at day 14. The activated astrocytes showed clear fluorescence of EphB2 in the SCI+cont shRNA group (C1–C4), but faint fluorescence of EphB2 in the SCI+EphB2 shRNA group (D1–D4). After 48 h LPS treatment, the primary cultured astrocytes were collected for Western blot assay to determine the expression of ephrin‐B2 and EphB2 (E). Histogram showed the expression of ephrin‐B2 (F) and EphB2 (G) proteins (*n* = 6, respectively). ELISA assay showed the expression of TGF‐β1 protein in the culture medium of astrocytes (H, *n* = 6). Scale bar = 25 µm, **p < 0.01, *t*‐test

Immunofluorescence showed low expression of ephrin‐B2 in astrocytes in the normal spinal cord (not at the injection point; Figure 2C1–4). However, the expression of ephrin‐B2 significantly increased after SCI on the activated astrocytes (Figure 4A1–4), and the expression remained high after RNAi (Figure 4B1–4).

In addition to the fibroblast expression of EphB2 at the injury site, the EphB2 fluorescent signal was visible on some cells after SCI (Figure 3A4,B4). Morphologically, these cells appeared to be activated astrocytes. Thus, immunofluorescence double staining of EphB2 and GFAP was performed. The activated astrocytes also showed clear EphB2 fluorescence (Figure 4C1–4), although not on the normal astrocytes (Figure 2E1–4). After RNAi, the activated astrocytes showed faint EphB2 fluorescence (Figure 4D1–4). To test the change in expression of EphB2 on activated astrocytes, we set up an in vitro LPS‐induced injury model. The ephrin‐B2 and EphB2 protein expression level was significantly increased in astrocytes after 48 h of exposure to LPS compared with the control group (*p* < 0.01; Figure 4E,F,G). We further examined the TGF‐β1 levels in the culture medium using ELISA kit. The data showed that the activated astrocytes secreted more TGF‐β1 than the control cells (*p* < 0.01; Figure 4H). In addition, it could also be seen from Figure 4 that many astrocytes expressed GFP, indicating that the shRNA successfully entered the astrocytes.

### 3.4 Electrophysiological parameters of animals at 3 m after SCI

**FIGURE 5:**
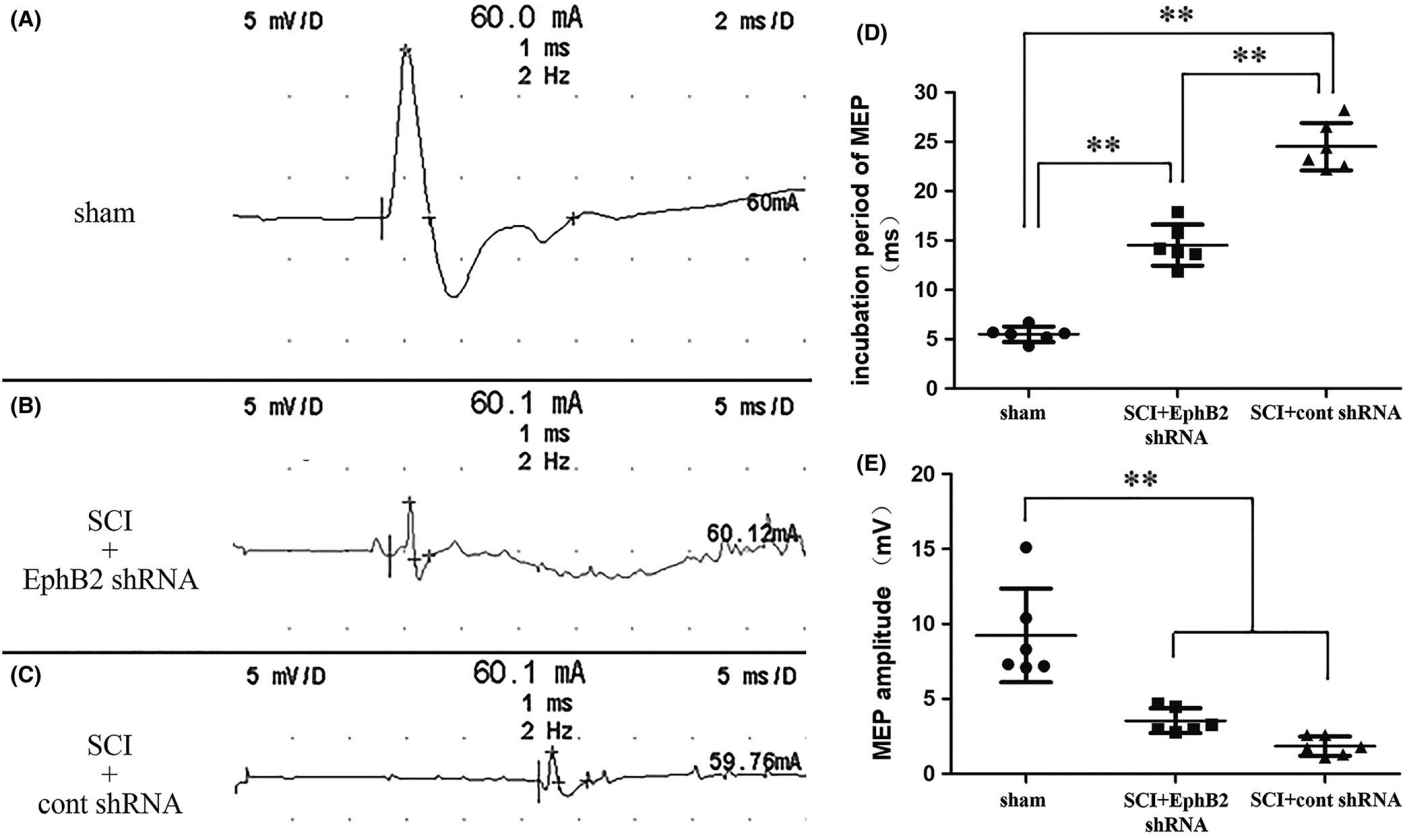
MEP examinations at 3 months after SCI. Representative recordings at sham (A), SCI+EphB2 shRNA (B), and SCI+cont shRNA group (C). Histograms showed the incubation period (D, *n* = 6) and MEP amplitudes (E, *n* = 6). **p < 0.01, one‐way ANOVA

The BBB motor behavioral test and CatWalk XT Automated Gait Analysis system was performed to detect the motor function of rats (seen in the Supplementary Materials). Although the data of the SCI+EphB2 shRNA group were slightly better than that of the SCI+cont shRNA group, there was no statistical difference (Figure S2). Subsequently, the MEPs were recorded at calf muscle (Figure 5A–C). The incubation period of MEP in the SCI+EphB2 shRNA group was significantly shorter than that in the SCI+cont shRNA group (*p* < 0.01; Figure 5D), but MEP amplitudes exhibited no significant difference between these two groups (Figure 5E).

### 3.5 Histological evaluation of regenerative nerve at 3 m after SCI

**FIGURE 6:**
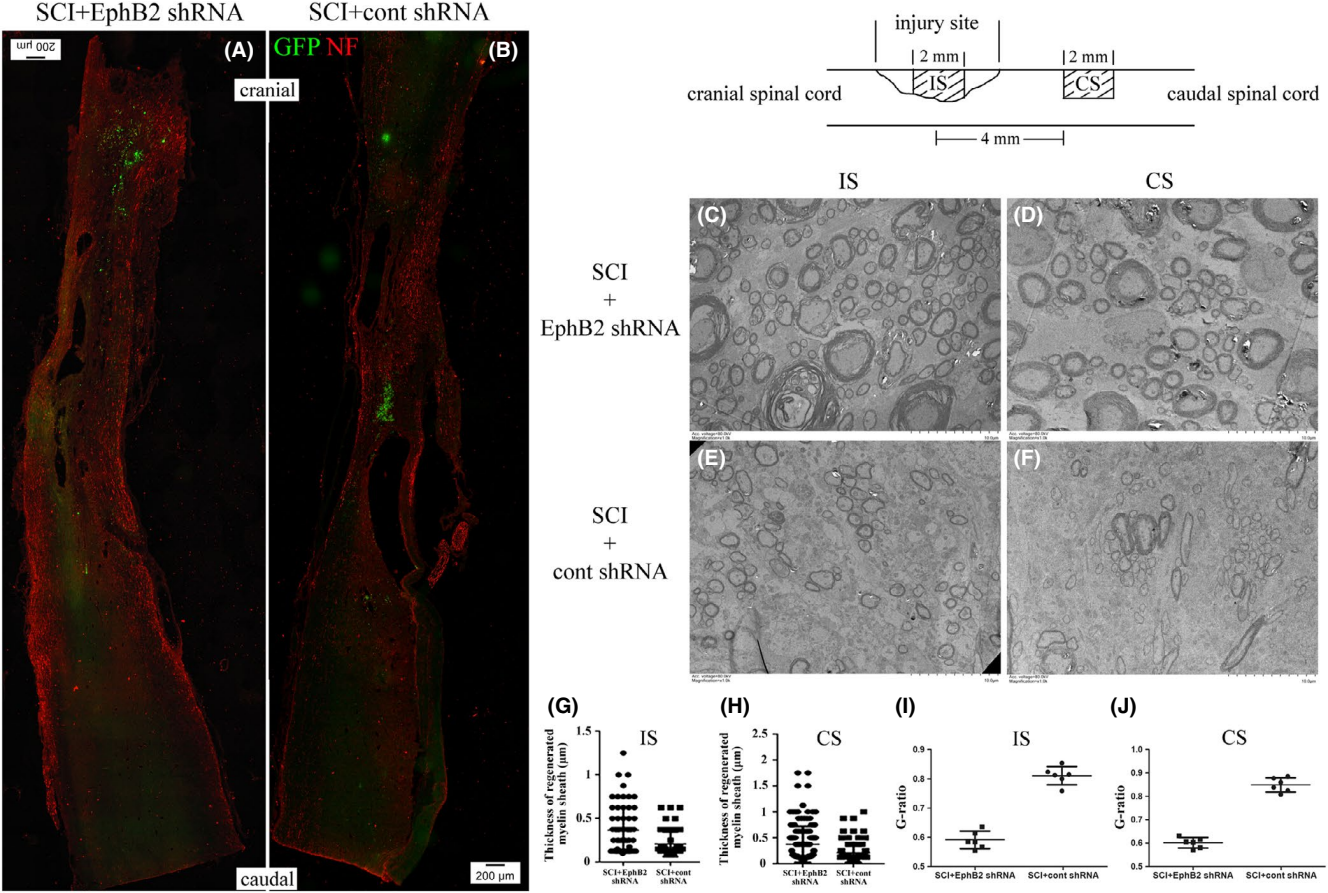
Regeneration of nerve fibers at 3 months after SCI. Anti‐NF immunohistochemistry of the longitudinal sectioned spinal cord in SCI+EphB2 shRNA (A) and SCI+cont shRNA group (B). Transmission electron micrographs of the IS and CS transverse section were taken from the spinal cord in SCI+EphB2 shRNA (C and D, respectively) and SCI+cont shRNA group (E and F, respectively). The diagram showed the distribution of the thickness of regenerated myelin sheath at IS (G) and CS (H) sites. The diagram showed the G‐ratio (myelinated axon diameter/myelinated fiber diameter) at IS (I) and CS (J) sites. Also shown (in the upper right inset) is the schematic diagram showing two sites. IS, injury site; CS, caudal spinal cord. Scale bar, 200 and 10 µm for (A, B) and (C–F), respectively

Anti‐NF immunohistochemistry and transmission electron microscope were used to observe the morphology of regenerated nerves. Three months after SCI, at the longitudinal sectioned spinal cord, more NF‐positive signals in SCI+EphB2 shRNA group (Figure 6A) entered the injury site and reached the caudal end compared with that in the SCI+cont shRNA group (Figure 6B). According to transmission electron microscopy, massive regenerated myelinated fibers displayed at the IS and CS taken from SCI+EphB2 shRNA group (Figure 6C,D) and exhibited clear, electron‐dense myelin sheath, and there were more large myelinated nerve fibers. On the contrary, at the IS and CS taken from SCI+cont shRNA group (Figure 6E,F), myelinated nerve fibers were fewer and thinner. The distribution of myelin thickness showed that the myelin sheath thickness in SCI+cont shRNA group was concentrated below 0.2 μm, but there were more thick myelin sheaths in SCI+EphB2 shRNA group (Figure 6G,H). The G‐ratio (myelinated axon diameter/myelinated fiber diameter) as a reliable measure was used to assess axonal myelination.^24^ Both G‐ratios of the SCI+cont shRNA group at the IS and CS were larger than that of the SCI+EphB2 shRNA group (Figure 6I,J). These suggested that the myelin sheaths in SCI+EphB2 shRNA group were more mature than SCI+cont shRNA group.

## 4 DISCUSSION

The formation of scar tissue is an inevitable process after SCI. Although the formation of glial scar is in favor of axon regeneration in the central nervous system (CNS),^25^ several latest reports show that decreased glial reaction and scar formation is beneficial to recovery from spinal cord injury.^26^, ^27^ After CNS injury, fibroblasts, pericytes, and inflammatory cells, in addition to astrocytes, participated in the formation of extracellular matrix.^10^ However, fibrotic scars and glial scars together built astroglial‐fibrotic scars, which had a negative effect on axon regeneration.^3^, ^28^, ^29^

Bundesen et al^13^ have found that after CNS injury, the expression of EphB2 receptor and its ephrin‐B2 ligand increased and that they bound to each other, resulting in accumulation of fibroblasts and astrocytes in the SCI margin layer and formation of extracellular matrix, and finally producing an astroglial‐fibrotic scar. Hereafter, several studies have confirmed this theory,^30^, ^31^, ^32^ which gave us the idea to try to inhibit the formation of scars from the beginning by interfering with EphB2 or ephrin‐B2, which are mainly expressed on fibroblasts or astrocytes, respectively. Ren et al^31^ published a report in which the formation of astroglial‐fibrotic scars in ephrin‐B2^−/−^ mice was obviously reduced after SCI. This study had the same purpose as our research design. They used target‐gene knockout technology, which is suitable for studying the effect of ephrin‐B2 and EphB2 on scar tissue formation. We, however, used RNAi to knock down the target gene, EphB2, and focused on elucidating the application possibility.

Many related articles have reported that EphB2 and ephrin‐B2 were upregulated to different degrees after SCI.^13^, ^30^, ^31^, ^32^ However, in some reports, after spinal nerve crush injury of SD rat, at different time points from 1 to 28 days, the expression of EphB2 was not detected on the spinal cord and dorsal root ganglion, and the expression of ephrin‐B2 was detected in the superficial dorsal horn of the spinal cord and dorsal root ganglia, and it was found on neurons rather than astrocytes.^21^ In this study, we used the rat model of spinal cord crush injury to further validate the location and expression levels of EphB2 and ephrin‐B2 after injury. The qPCR results showed that the expression of EphB2 and ephrin‐B2 increased in a time‐dependent manner after SCI. In our experiment, the high expression of EphB2 and ephrin‐B2 mainly appeared on the astroglial‐fibrotic scars. The difference from the results reported by Kobayashi et al^21^ may be due to the different models of nerve injury. It has been reported that complete transverse spinal cord injuries result in larger astroglial‐fibrotic scars and a greater number of fibroblasts in the injury area.^13^ However, in the contusion injury in this experiment, fibroblasts also invaded into the injury area. Although the number of fibroblasts was relatively small, they could form astroglial‐fibrotic scars together with astrocytes.

It is generally believed that at 14 days after SCI, the formation of astroglial‐fibrotic scars is stable^13^; hence, we selected this time point for observing the effect of RNAi. After the vector viruses infected the related cells, the specific shRNA suppressed EphB2 expression, whereas the expression of ephrin‐B2 was not affected. From a morphological perspective, the fluorescence intensity of EphB2 and FN was reduced at the injury site after RNAi. Moreover, the dense expression of GFAP was not obvious at the injury border after RNAi. These results indicated that the RNAi of EphB2 had not only affected fibroblast activation, proliferation, and invasion into the depths of the SCI but also affected the migration of astrocytes at the injury border. Finally, it blocked the binding between fibroblasts and astrocytes to form astroglial‐fibrotic scars. Therefore, exactly as the NF immunofluorescence staining and electron microscopy showed, this effect was beneficial for regenerating axons through the injured area by inhibiting the expression of EphB2.

Our results demonstrated that the activated astrocytes expressing ephrin‐B2 also highly expressed EphB2 in vivo and in vitro, which is different from previous report.^13^ It has been reported that Eph receptors and ephrins can be co‐expressed in the same cells.^33^ Eph receptors and ephrins engage in a multitude of activities, including cell morphology, adhesion, migration, proliferation, survival, and differentiation.^33^ Similarly, our in vitro experiments showed that LPS‐activated astrocytes secreted more TGF‐β1 than the control group. After CNS injury, TGF‐β1 produced and released by astrocytes increases around the lesion site, to enhance fibroblast proliferation.^34^, ^35^, ^36^ Therefore, we think that the activated astrocytes expressing ephrin‐B2 and the fibroblasts expressing EphB2 mainly together formed the astroglial‐fibrotic scar; in the same way, the astrocytes expressing EphB2 may interact with astrocytes expressing ephrin‐B2 and also participate in the formation of glial scars. We speculate that knocking down EphB2 inhibited astrocyte aggregation in the surrounding region of the injury, decreased astrocyte‐derived TGF‐β1 secretion, and further suppressed proliferation of fibroblasts and the formation of astroglial‐fibrotic scars. However, this hypothesis needs to be confirmed by additional experiments. Moreover, because of the large proportion of astrocytes in the astroglial‐fibrotic scar, more viruses carrying lentiviral vectors containing shRNA that targets EphB2 and the ZsGreen1 reporter gene entered astrocytes than fibroblasts, and therefore, EphB2 expression was significantly inhibited in the former than in the latter.

Although RNAi technology can knock down the overexpression of EphB2 after SCI and effectively control the formation of astroglial‐fibrotic scars, which was beneficial for axon regeneration, behavioral and electrophysiological assessments demonstrated that functional recovery was not ideal. It suggests that full SCI repair cannot simply rely on this method. There are multiple aspects of SCI pathology that need to be addressed. Efforts have been made on testing the therapeutic effects of neurotrophic factors, cell transplantation, removal of inhibitory molecules, tissue engineering, neurorehabilitation, and others, with varying levels of outcomes; however, none of these treatment attempts, when employed separately, led to optimal functional recovery.^37^ Because the orientation of various fiber tracts in the spinal cord is extremely complex, how to direct the regenerated axons to their correct targets, including the reinnervation of the motor neurons that can prevent muscular atrophy, is critical for the recovery of function. This may also explain why the functional recovery was limited while there were a substantial number of regenerating nerve fibers in the present study. Therefore, combinational strategies may be achieved better therapeutic effects,^37^ as shown by our group recently.^38^

## CONFLICT OF INTEREST

We have not published or submitted the manuscript elsewhere simultaneously. The authors taking part in this study declared that they do not have any conflict of interests in this manuscript.

## Supporting information

Figure S1

Figure S2

Supplementary Material

## Data Availability

The authors confirm that the data supporting the findings of this study are available within this article and from the corresponding author upon reasonable request.
