# Supplementary figures and images for: EphB2 knockdown decreases the formation of astroglial‐fibrotic scars to promote nerve regeneration after spinal cord injury in rats
Source: CNS Neurosci Ther. 2021 Apr 1;27(6):714–24. doi: 10.1111/cns.13641 (PMC8111500; doi:10.1111/cns.13641)

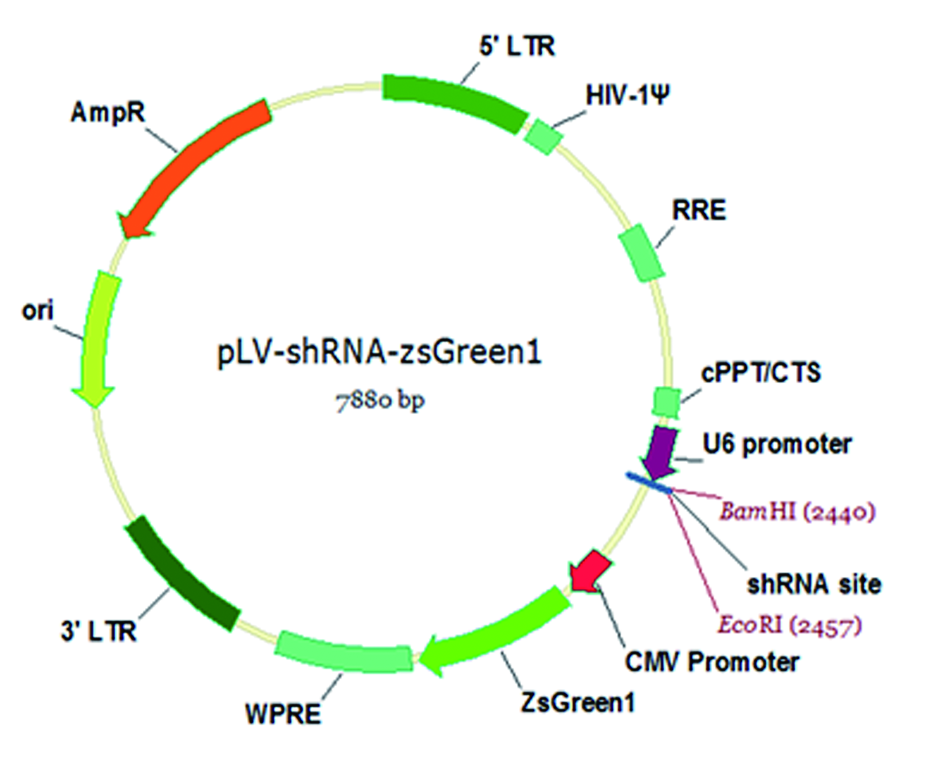

Supplement: Supplementary file 1 — Figure S1 [file CNS-27-714-s002.tif]

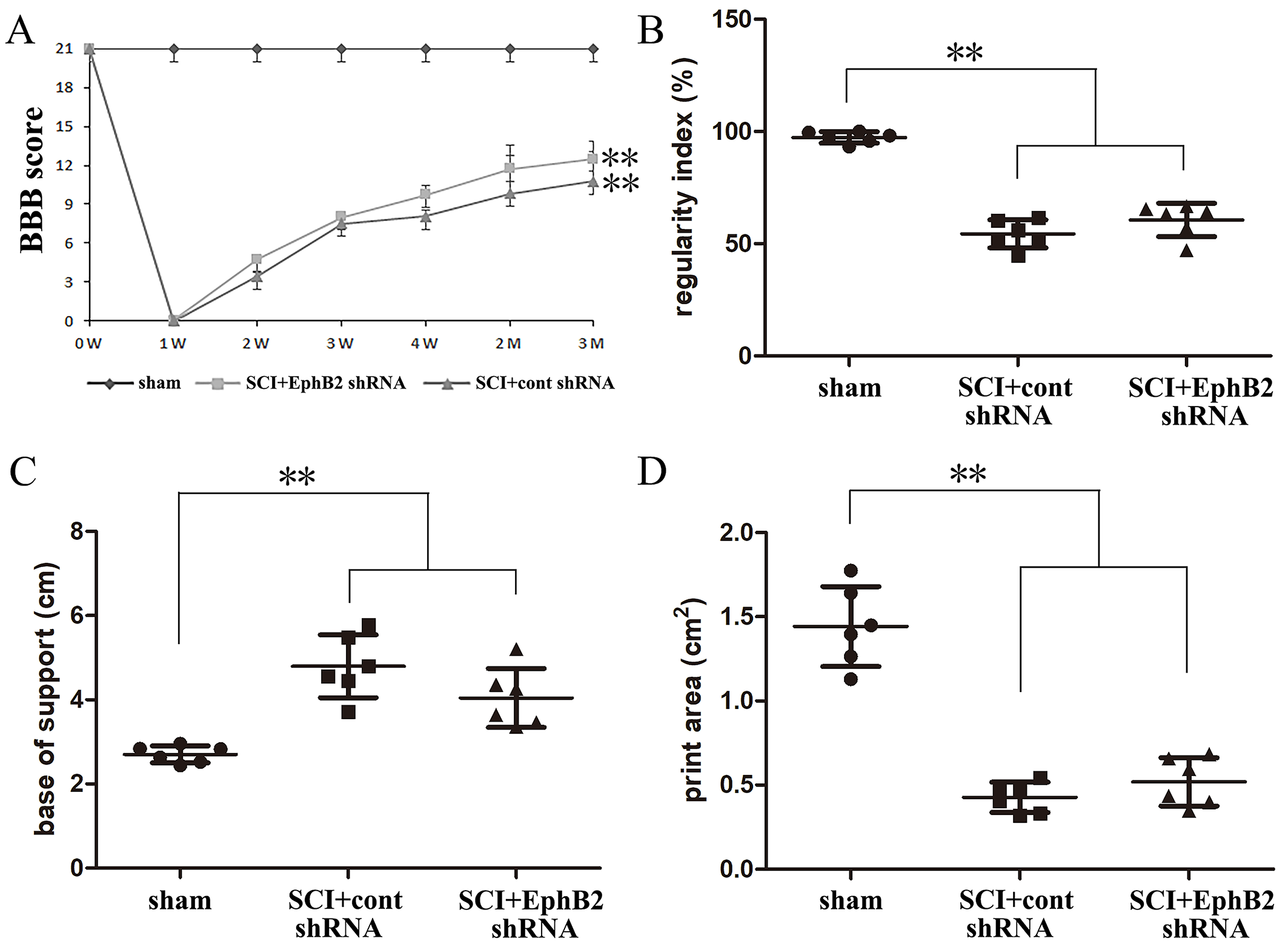

Supplement: Supplementary file 2 — Figure S2 [file CNS-27-714-s001.tif]
