# Supplementary material for: EphB2 knockdown decreases the formation of astroglial‐fibrotic scars to promote nerve regeneration after spinal cord injury in rats
Source: CNS Neurosci Ther. 2021 Apr 1;27(6):714–24. doi: 10.1111/cns.13641 (PMC8111500; doi:10.1111/cns.13641)
Supplement: Supplementary file 3 — Supplementary Material [file CNS-27-714-s003.docx]

**Supplementary Materials and Methods**

**Animal Models**

First, the rats were injected intraperitoneally (2.5 mL / kg) with a mixture of ketamine (62.50 mL / kg), xylazine (3.18 mL / kg), and acepromazine (0.63 mL / kg). An incision was made on the dorsal aspect of the spine from T8 to T10, and the lamina was opened to expose the spinal cord. In the Sham group, the rats were not subjected to spinal injury and were sutured directly from the inside to the outside. In the rest of the groups, the spines were fixed, and then the spinal cord was hit (1.5 N) using an IH-0400 impactor (Precision Systems and Instruments, Fairfax Station, VA, US). In the SCI+EphB2 shRNA group and the SCI+cont shRNA group, the rats were immediately injected with the shRNA virus for *EphB2* or scrambled shRNA, using a stereotaxic apparatus and a Quintessential Stereotaxic Injector (Stoelting, Wood Dale, IL, US). Because the diameter of the head of impact was 2 mm, after measurement, the center and peripheral region of the apparent tissue injury formed by the impact was within 2.1 mm and the maximum depth was about 1.8 mm at the center of injury. To inject the virus at the SCI boundary, where the astroglial-fibrotic scar forms, the injection site was at the center of injury, 2 mm away from the cranial and caudal levels, with a depth of 1.5 mm. The injection speed was 0.3 µL/min and the virus titer was 2×10^7^ TU/mL, retaining the needle for 2 min, and then the incision was closed. After surgery, each rat immediately put in a warm room to restore normal body temperature, injected intraperitoneally with 100,000 units of penicillin once a day for 3 d, and the bladder was massaged twice a day for voiding until the rats were able to urinate autonomously.

**Behavioral testing**

All rats walked freely in an open field, and were observed for 5 min. Basso, Beattie, and Bresnahan (BBB) scores were measured as described (Basso et al., 1996) at 0, 1, 2, 3, 4 w, 2 and 3 m after SCI.

Three months after SCI, gait analyses were performed using the CatWalk XT 9.0 system (Noldus, Wageningen, Netherlands) as described in previous reports (Hamers, Koopmans, & Joosten, 2006). Three separate runs per animal were performed, and the data were processed and analyzed by the CatWalk software. We selected the results commonly used in research on SCI (Hamers et al., 2001), such as regularity index, base of support, and print area.

**Quantitative PCR and western blot** **analyses**

qPCR: PCR primers (Table S1) were synthesized by Invitrogen. The extracted RNA was amplified using the StepOne™ real-time fluorescent quantitative PCR system (Applied Biosystems, Carlsbad, CA, USA). The results were analyzed using *β-actin* as an internal reference.

Western blotting: the extracted proteins were separated by 8% or 10% SDS-PAGE and transferred to polyvinylidene fluoride membranes. After blocking with 5% skim milk in TBST for 2 h, the membranes were incubated with primary antibodies (Table 1) overnight at 4°C. After three washes with TBST, the membranes were incubated with the secondary antibodies (Table 1) at room temperature for 2 h. After three washes, the membranes were visualized using enhanced chemiluminescence (Millipore, Darmstadt, Germany). Finally, the intensity of each band was determined by using Imagelab 5.1 software (Bio-Rad Laboratories Inc., Hercules, CA, USA).

**Supplementary Tables**

Table S1. PCR Primer

| Gene name | Positive-sense strand | Antisense strand |
| --- | --- | --- |
| EphB2 | 5'-GAGGTGAGCGGCTACGAT-3' | 5'-TTGGGAAAGGTCTTGGTG-3' |
| ephrin-B2 | 5'-ACCGCTAAGGACTGCAGACAG-3' | 5'-GTCCAAGTGGGGATCTCCTAG-3' |
| β-actin | 5'-AGGCATCCTGACCCTGAAGTAC-3' | 5'-TCTTCATGAGGTAGTCTGTCAG -3' |

**Figure S legend**

Figure S1. shRNA information based on lentiviral vectors (pLV-shRNA-zsGreen1).

Figure S2. Locomotor function assessed with BBB score and CatWalk XT Automated Gait Analysis system.

(A) BBB scores were measured at 0, 1, 2, 3, 4 w, 2 and 3 m after SCI. (B–D) Three parameters of CatWalk assessment were selected to analyze recovery at 3 m after SCI: regularity index, base of support, and print area. **p < 0.01 versus sham group.
